# Force Steadiness During a Cognitively Challenging Motor Task Is Predicted by Executive Function in Older Adults

**DOI:** 10.3389/fphys.2018.01316

**Published:** 2018-10-02

**Authors:** Hugo M. Pereira, Bonnie Schlinder-Delap, Kristy A. Nielson, Sandra K. Hunter

**Affiliations:** ^1^Department of Health and Exercise Science, University of Oklahoma, Norman, OK, United States; ^2^Exercise Science Program, Department of Physical Therapy, Marquette University, Milwaukee, WI, United States; ^3^Department of Psychology, Marquette University, Milwaukee, WI, United States

**Keywords:** force fluctuations, sex differences, gender differences, aging, Trail-making Test

## Abstract

Motor performance and cognitive function both decline with aging. Older adults for example are usually less steady for a constant-force task than young adults when performing low-intensity contractions with limb muscles. Healthy older adults can also show varying degrees of cognitive decline, particularly in executive function skills. It is not known, however, whether age-related changes in steadiness of low-force tasks and cognitive function are independent of one another. In this study, we determined if executive function skills in aging are associated with the steadiness during a low-force muscle contraction performed with and without the imposition of a cognitive challenge. We recruited 60 older adults (60–85 years old, 34 women, 26 men) and 48 young adults (19–30 years old, 24 women, 24 men) to perform elbow flexor muscle contractions at 5% of maximal voluntary contraction (MVC) force in the presence and absence of a difficult mental-math task (counting backward by 13 from a four-digit number). Force steadiness was quantified as the coefficient of variation (CV) of force and executive function was estimated with the Trail-making Test part A and B. The cognitive challenge increased the CV of force (i.e., decreased force steadiness) with greater changes in older adults than young adults (5.2 vs. 1.3%, respectively, cognitive challenge × age: *P* < 0.001). Older adults were 35% slower in both parts A and B of the Trail-making Test (*P* < 0.001), and to eliminate the effects of age and education on this variable, all further analyses were performed with the age-corrected *z*-scores for each individual using established normative values. Hierarchical regression models indicated that decreased force steadiness during a cognitive challenge trial was in part, explained by the performance in the Trail-making Test part A and B in older (*r* = 0.53 and 0.50, respectively, *P* < 0.05), but not in young adults (*P* > 0.05). Thus, healthy community-dwelling older adults, who have poorer executive function skills, exhibit reduced force steadiness during tasks when also required to perform a high cognitive demand task, and are likely at risk of reduced capacity to perform daily activities that involve cognitively challenging motor tasks.

## Introduction

Many activities of daily living require precise control of force during static or dynamic contractions (e.g., eating, cooking, and interacting with touch screen devices). Most of these motor tasks require not only an intact musculoskeletal system, but they also depend upon integral neural function (Scherder et al., 2008; Overdorp et al., 2016). Cognitive processes such as attention, memory, and executive function are essential neural constructs often involved during motor tasks (Spirduso, 1980; Scherder et al., 2008; Corp et al., 2013). More specifically, executive function which encompasses working memory and attention, enables an individual to plan, organize, and integrate neural processes to achieve a goal (Elliott, 2003). Executive functioning, for example, is essential during activities of daily living that require divided attention (i.e., dual task activities) because a dual task often requires planning, integration of information and the capacity to switch attention between tasks (Strauss et al., 2006).

It is well established that both motor and cognitive function decline with aging (Buckner, 2004; Salthouse, 2009; Hunter et al., 2016). In the laboratory setting, motor function and the resultant force fluctuations (force steadiness) can be quantified for a force-matching task of a muscle group as the variability of the force around the mean force [Coefficient of variation of force (CV) = standard deviation/mean × 100] (Enoka et al., 2003). Using this metric older adults and women frequently exhibit a greater CV of force (reduced force steadiness) compared with young adults and men, respectively, at lower intensities of contraction (Tracy and Enoka, 2002; Enoka et al., 2003; Brown et al., 2010). Greater maximal strength can partially account for some of the reduced CV of force with aging in men and women (Christou and Carlton, 2001; Marmon et al., 2011).

Force steadiness, however, is altered with the requirement of greater cognitive demand during the motor task. Imposing a cognitive challenge during a force-matching motor task, for example, resulted in greater CV of force in older adults, particularly older women, for both upper and lower limb muscles (Voelcker-Rehage et al., 2006; Vanden Noven et al., 2014; Pereira et al., 2015; Shortz and Mehta, 2017), although the mechanism is not well defined. We also found larger variability between trials in motor performance among older men and women compared with young adults when a cognitive challenge was imposed during a low-force, steadiness task for the lower limb (Vanden Noven et al., 2014). Furthermore, when the difficulty in the cognitive challenge increased, steadiness reduced (i.e., CV of force increased) markedly for the older adults and less for the young (Voelcker-Rehage et al., 2006; Vanden Noven et al., 2014; Pereira et al., 2015). One possible explanation is that older men and women who have age-related reductions in executive functions, have lower capacity to multi-task and thus poorer performance on a motor task while also performing a cognitive challenge. However, the influence of baseline cognitive constructs, such as executive function, on motor function such as a task that requires force steadiness in young and older adults are poorly understood. One of the several executive function tests available to target this construct is the Trail-making Test (Reitan, 1958; Tombaugh, 2004) (see better description in the section “Materials and Methods”), which is frequently used in neuropsychological batteries (Strauss et al., 2006).

The purpose of the study therefore, was to determine if executive functioning, estimated with the Trail-making Test, was associated with force steadiness (CV of force) during an upper extremity task both with and without the imposition of cognitive challenge in young and older men and women. Our *hypothesis* was that poorer executive functioning would predict the CV of force during a motor task when performed simultaneously with a cognitive challenge because of the greater reliance on executive functioning during the increased cognitive demand task.

## Materials and Methods

Sixty older (60–85 years old, 34 women and 26 men) and 48 young adults (19–30 years old, 24 women and 24 men) participated in the study (**Table 1**). Each participant provided written informed consent to participate in the study and the protocol was approved by the Institutional Review Board of Marquette University and all experimentations were performed in accordance with the Declaration of Helsinki. All participants were healthy without known neurological, orthopedic or cardiovascular conditions and they were naive to the protocol. All older women were post-menopausal and none were on hormone replacement therapy at the time of the study.

**Table 1:** Descriptive statistics [mean (SD)] of young and older men and women.

	Young men	Young women	Old men	Old women
	
*n*	24	24	26	34
Age (years)	22.1 (3.1)	21.6 (2.6)	69.6 (5.5)	68.2 (6.4)
Education (years)	16.2 (2.3)	15.6 (1.9)	16.8 (2.7)	15.7 (3.5)
GDS (a.u.)	–	–	1.22 (1.3)	1.7 (1.9)
MMSE (a.u.)	–	–	28.5 (1.6)	28.9 (1.6)
Trait anxiety (a.u.)	35.7 (8.2)	32.7 (8.6)	30.2 (6.8)	28.9 (6.4)
PAQ (MET-h/week)	66.1 (56.6)	55.6 (47.8)	37.5 (30.9)	31.5 (23.1)
Handedness (a.u)	0.6 (0.4)	0.7 (0.2)	0.8 (0.3)	0.7 (0.4)
MVC (N.m)	71.5 (19.1)*	42.3 (10.0)	62.2 (12.7)^†^	33.1 (6.4)
Trails A (s)	15.7 (3.3)	16.7 (5.8)	23.9 (6.1)	23.1 (6.8)
Trails B (s)	35.5 (11.6)	35.9 (9.3)	58.5 (23.4)	55.8 (20.6)

*GDS, Geriatric Depression Scale short version raw score (possible range = 0–15); MMSE, Mini Mental State Exam raw score (possible range = 0–30); Trait Anxiety, State-Trait Anxiety Inventory raw score (possible range = 20–80); PAQ, Physical Activity Questionnaire; Handedness, Edinburgh Handedness Inventory [range = -1 (left) to +1 (right)]; MVC, maximal voluntary contraction; Trails, Trail-making Test; ^∗^sex difference for young adults; ^†^sex difference for older adults.*

Each participant attended three sessions, an introductory session and two randomized experimental sessions. At the introductory session, each participant completed surveys to evaluate handedness (Oldfield, 1971), physical activity levels (Kriska and Bennett, 1992), and trait anxiety (Spielberger et al., 1970). To screen for dementia and depression, each older individual completed the Mini-Mental State Examination (Folstein et al., 1975) (all participants scored >24) and the Geriatric Depression Scale (Sheikh and Yesavage, 1986) (all participants scored <5). Executive functioning was measured using the Trail-making Test, and each participant was familiarized with the steadiness task as described later.

In separate experimental sessions, each participant performed a: (*a*) *control trial*, which involved performance of a force steadiness task with the elbow flexor muscles *without* imposition of a mental math task (cognitive challenge) and, (*b*) *cognitive challenge trial*, which involved a force steadiness task with the elbow flexor muscles while also performing a mental math task (cognitive challenge). The force steadiness task during each session was performed with the elbow flexor muscles at 5% of maximal voluntary contraction (MVC) force and the order of the sessions (control and cognitive challenge trials) was randomized.

### Assessment of Executive Functioning: Trail-Making Test

The Trail-making Test (Reitan, 1958) is comprised of two parts: In part A, each participant was asked to draw a line consecutively connecting 25 encircled numbers distributed on a sheet of paper. In part B, each circle had either a number or a letter in its center, and each participant was asked to consecutively connect the circles alternating between numbers and letters (e.g., 1, A, 2, B, 3, C, 4, D, and so on), as fast as possible while maintaining accuracy. The score on each part is the time required to connect the circles without removing the pen from the paper.

Although parts A and B of the Trail-making Test are highly intercorrelated, they are differentially affected by aging (Tombaugh, 2004) due to differences in the cognitive constructs they assess (Bowie and Harvey, 2006). Specifically, part B imposes greater cognitive demand and need for flexibility by requiring switching between letters and numbers, whereas part A more simply requires the ability to maintain a cognitive set (Kortte et al., 2002). Part B measures executive control and it is less influenced by motor components, whereas part A is generally associated with attention and motor speed (Arbuthnott and Frank, 2000). Consequently, we analyzed parts A and B separately to investigate any potential distinct effects between tests when a motor task is performed simultaneously with a cognitive challenge.

### Assessment of the Force Steadiness

Each participant was seated upright in an adjustable chair with the non-dominant arm abducted slightly and the elbow resting on a padded support with the elbow joint flexed to 90°. The non-dominant arm was tested to minimize variability between participants that can occur due to differences in activities performed with the dominant arm. Details of the experimental setup are described elsewhere (Pereira et al., 2015). In brief, the hand and forearm were placed in a modified wrist–hand–thumb orthosis (Orthomerica, Newport Beach, CA, United States), and the forearm was placed midway between pronation and supination. Elbow flexion force was measured with a transducer (JR-3 Force-Moment Sensor; JR-3, Woodland, CA, United States, range ± 800 N; resolution: 0.10 N or MLP – 150 Transducer Techniques, Temecula, CA, United States, resolution: 0.10 N securing that force signals were similar between transducers) and displayed on a 22′′ monitor. Force was recorded online at 500 samples/s using a Power 1401 analog-to-digital (A–D) converter and Spike 2 software [Cambridge Electronic Design (CED), Cambridge, United Kingdom].

The following protocol was followed to assess force steadiness:

(1) *Assessment of Maximal Voluntary Contraction (MVC).* Each participant performed 3–4 MVCs trials with the elbow flexor muscles with 60 s rest between each trial. If the peak force achieved for two of the first three trials was not within 5% of each other, additional trials were performed until this criterion was met. The greatest force achieved with the elbow flexor muscles was taken as the MVC force and used to calculate the target force for the submaximal contractions.

(2) *Submaximal Contractions.* After the MVC was determined, an isometric contraction at 5% of MVC was performed for 40 s. A very low intensity of 5% MVC was chosen because previous studies indicated that age differences in the force steadiness with cognitive challenge are more likely at very low contraction intensity (Pereira et al., 2015; Hunter et al., 2016). During the cognitive challenge trial, once the participant increased the force to the required target force, the participant began the subtraction by 13 from a four-digit number. Only one trial was performed during the experimental session because the learning effect was minimal in a subset of participants and also to simplify the test session. During the control trial, each participant performed the submaximal contraction only.

### Cognitive Challenge Task

The cognitive challenge involved mental math, such that each individual performed serial subtraction by 13 from a four-digit number with one response required every 3 s (Noteboom et al., 2001; Pereira et al., 2015). If the participant made an error in the serial subtraction or was unable to provide the correct answer within 3 s, the mental-math procedure was restarted with a new number (Noteboom et al., 2001; Yoon et al., 2009; Keller-Ross et al., 2014; Vanden Noven et al., 2014; Pereira et al., 2015). The cognitive challenge was performed at two timepoints: (1) before the submaximal contraction for 4 min for practice, and (2) during the steadiness task in a dual task. Mental math was performed during the cognitive challenge trial only, and not during the control trial.

### Data Analysis

#### Analysis of the Trail-Making Test

Older adults are known to take a longer duration to complete the Trail-making Test (Tombaugh, 2004). Thus, age itself could serve as a confounding factor when determining the influence of executive functioning on force steadiness. Norms for the Trail-making Test are stratified by age and education to correct for the influence of both factors on performance (Tombaugh, 2004). Thus, we calculated Trail-making *z*-scores accounting for age and education to minimize their potential impacts on the results. Average raw scores for each group are presented in **Table 1**.

#### Analysis of the Force Signal During MVC and the Steadiness Task

The torque was calculated as the product of force and the distance between the elbow joint and the point at which the wrist was attached to the force transducer. The MVC was quantified as the average value over a 0.5 s interval that was centered about the peak. Force steadiness was quantified with the amplitude of the force fluctuations using the coefficient of variation of the force (CV = standard deviation of the force/mean of force × 100). The CV of force was calculated over the middle 30 s period of each 40 s submaximal contraction.

### Statistical Analysis

Data are reported as mean ± SD within the text and tables and mean ± SE in the figure. CV of force and MVC force were analyzed with repeated measures analysis of variance (ANOVA) with age and sex as between-subject factors. Repeated measures included the test trial (control vs. cognitive challenge). Separate two-factor (age × sex) ANOVAs were used to compare handedness, physical activity levels, Trail-making Test part A and B, trait of anxiety and years of education between young and older men and women. For each ANOVA the sphericity of data was verified with *Mauchly’s* test. In cases where *F*-test was significant, *post hoc t*-tests with *Bonferroni* corrections were performed to detect differences among pairs. Independent *t*-tests were used to compare the results of Mini-Mental State Examination and Geriatric Depression Scale between men and women in the older adults group after assessing for normality with the *Shapiro–Wilk* test. Hierarchical regression models were used to determine the influence of the executive functioning on the CV of force in the control trial and the cognitive challenge trial. Age, sex, and MVC were entered as predictors in the first step, as each has known potential influence on the CV of force (the criterion variable). Trail-making Test performance part A and B were entered as predictors in step 2 (independent models as different indices of executive functioning). The statistical significance was considered as *P* < 0.05 and all analysis were performed in IBM Statistical Package for Social Sciences (SPSS) version 23.

## Results

Young adults were stronger than older adults (age effect: *P* < 0.01) and men were stronger than women (sex effect: *P* < 0.01), with no interaction of age and sex (*P* = 0.99, **Table 1**). Young adults reported greater physical activity levels compared with older adults (59.4 ± 50.5 vs. 33.2 ± 26.2 MET-h/week, respectively, age effect: *P* < 0.001), with no sex differences (sex effect: *P* = 0.39) and no interaction of age and sex (*P* = 0.54). There were no differences in handedness, years of education and trait of anxiety across groups (all *P* > 0.05) (**Table 1**).

### Steadiness

**FIGURE 1:**
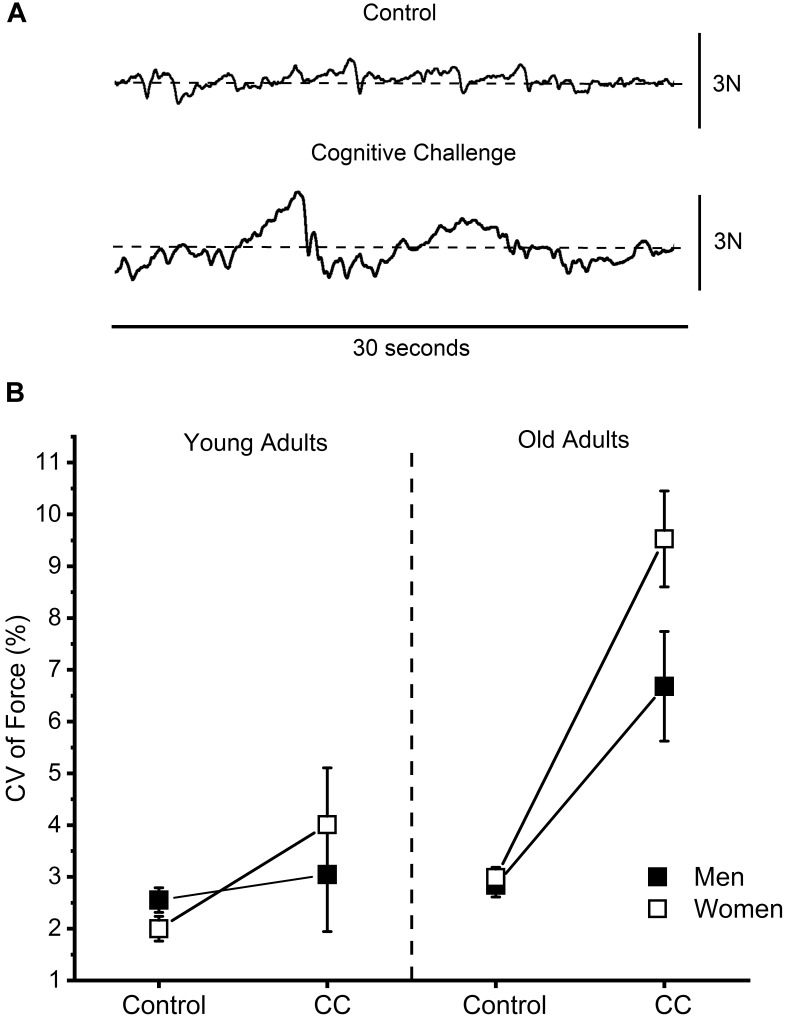
**(A)** Representative force signals of the elbow flexor muscles for control and cognitive challenge trials (subtracting by 13 from a four-digit number). **(B)** Force steadiness quantified as the coefficient of variation (CV) of force during the control and cognitive challenge trials (CC) of young and older men and women. Older adults had a greater increase in CV of force (i.e., reduced force steadiness) during the CC trials than young adults (*P* < 0.001) and women had a greater increase in CV of force during the CC trials than men (*P* = 0.03).

Older adults had greater CV of force (i.e., reduced steadiness) compared with the young adults (5.5 ± 4.1% vs. 2.9 ± 1.6%, respectively, age effect: *P* < 0.01) and women had greater CV of force than men (6.3 ± 3.6% vs. 4.2 ± 3.7%, respectively, sex effect: *P* = 0.01) with a trend for greater CV of force in older women (age × sex: *P* = 0.06). CV of force was greater during the cognitive challenge trial compared with the control trial (5.8 ± 7.7% vs. 2.6 ± 1.7%, respectively, cognitive challenge effect: *P* < 0.001) (**Figure 1A**), but the older adults had a greater increase in CV of force between the control to the cognitive challenge trial (control: 2.9 ± 1.2% vs. cognitive challenge trial: 8.1 ± 5.4%) than young adults (control: 2.2 ± 1.2% vs. cognitive challenge trial: 3.5 ± 6.0%; cognitive challenge effect × age: *P* < 0.001). Women had a greater increase in CV of force from the control to the cognitive challenge trial (2.5 ± 1.2% vs. 6.8 ± 5.5%, respectively) than the men (2.7 ± 1.2% vs. 4.9 ± 5.4%, respectively, session × sex: *P* = 0.03) for both young and older adults (session × age × sex: *P* = 0.57) (**Figure 1B**).

### Trail-Making Test (Raw Scores)

#### Part A

Older adults required a longer time to complete the test compared with young adults (25.3 ± 6.2 vs. 16.6 ± 6.3 s, respectively, age effect: *P* < 0.001), with no difference between men and women (sex effect: *P* = 0.39) in either the young or older adults (age × sex: *P* = 0.43, **Table 1**).

#### Part B

Time to complete the test was longer for the older adults than young adults (58.3 ± 23.7 s vs. 36.3 ± 10.5 s, respectively, age effect: *P* < 0.001), with no difference between men and women (sex effect: *P* = 0.60) in either the young or older adults (age × sex: *P* = 0.73, **Table 1**).

### Predictability of Force Steadiness

Hierarchical regression models were used to identify variables that influenced the CV of force during the control and cognitive challenge trials (**Tables 2**, **3**). Step 1 showed the effects of age, sex and MVC on the CV of force, and step 2 showed the added contribution to prediction of CV by the *z*-score results for the Trail-making Test (parts A and B, independently) for young (**Table 2**) and older adults (**Table 3**).

**Table 2:** Hierarchical regression analyses predicting performance on force steadiness of young adults.

		Model summary of each step					Contribution of each variable in last step				
		*R*	*R^2^*	Δ*R*^2^	*F*	*p*	B	SE	β	*t*	*p*
**Force steadiness: control trial**						
Step 1		0.49	0.24	–	4.33	**0.01**					
	Age						0.02	0.06	0.04	0.28	0.78
	Sex						-1.52	0.45	-0.62	-3.36	**<0.01**
	MVC						-0.04	0.01	-0.59	-3.21	**<0.01**
Step 2		0.52	0.27		1.91	0.17					
	Trails A						0.31	0.22	0.18	1.38	0.17
Step 2		0.49	0.24	<0.01	0.41	0.53					
	Trails B						-0.13	0.21	-0.09	-0.64	0.53
**Force steadiness: cognitive challenge trial**						
Step 1		0.34	0.12	–	1.91	0.14					
	Age						0.19	0.11	0.25	1.72	0.09
	Sex						1.00	0.81	0.25	1.23	0.23
	MVC						-0.01	0.02	-0.05	-0.23	0.82
Step 2		0.36	0.13	0.05	0.21	0.65					
	Trails A						0.19	0.40	0.07	0.46	0.65
Step 2		0.40	0.16	0.04	1.73	0.20					
	Trails B						0.48	0.36	0.19	1.32	0.20

*MVC, maximal voluntary contraction; Trails, Trail-making Test; F from change in R^*2*^, P from change in R^*2*^. Bold values highlight when *P* < 0.05 (i.e., statistically significant).*

**Table 3:** Hierarchical regression analyses predicting performance on force steadiness of older adults.

		Model summary of each step					Contribution of each variable in last step				
		*R*	*R*^2^	Δ*R*^2^	*F*	*p*	B	SE	β	*t*	*p*
**Force steadiness: control trial**						
Step 1		0.42	0.18	–	3.98	**0.01**					
	Age						0.05	0.02	0.25	1.95	0.05
	Sex						-0.74	0.60	-0.31	-1.25	0.22
	MVC						-0.04	0.02	-0.53	-2.14	**0.03**
Step 2		0.45	0.2	0.14	1.71	0.19					
	Trails A						0.28	0.22	0.16	1.31	0.19
Step 2		0.46	0.21	0.04	2.39	0.13					
	Trails B						0.27	0.18	0.19	1.55	0.13
**Force steadiness: cognitive challenge trial**						
Step 1		0.42	0.18	–	3.98	**0.01**					
	Age						0.44	0.16	0.36	2.82	**0.01**
	Sex						3.13	3.58	0.22	0.88	0.39
	MVC						-0.02	0.11	-0.06	-0.23	0.82
Step 2		0.53	0.28	0.11	7.45	**<0.01**					
	Trails A						3.32	1.22	0.32	2.73	**0.01**
Step 2		0.50	0.25	0.07	4.98	**0.03**					
	Trials B						2.30	1.03	0.27	2.23	**0.03**

*MVC, maximal voluntary contraction; Trails, Trail-making Test; F from change in R^*2*^, P from change in R^*2*^. Bold values indicate when *P* < 0.05 (i.e., when they are statistically significant).*

#### Control Trials

The age of the participant did not predict the CV of force for the young (*P* = 0.78) (step 1 in **Table 2**) but there was a trend for the older adults (*P* = 0.05) (step 1 in **Table 3**). The sex of the participant did not predict the CV of force during control trials for the older adults (*P* = 0.22) (step 1 in **Table 3**) but did explain the CV of force in young adults (*P* < 0.01) (step 1 in **Table 2**). Greater MVC was responsible for 0.59 and 0.53% reduction in the CV of force (revealed by the beta values) for young and older adults, respectively (both groups with *P* < 0.05) (step 1 in **Tables 2**, **3**). Including the *z*-score result of the Trail-making Test part A did not alter the prediction of CV of force for young or older adults (both with *P* > 0.05) (step 2 in **Tables 2**, **3**). This was similar for the Trail-making Test part B *z*-score results for the young and older adults (both groups with *P* > 0.05) (step 2 in **Tables 2**, **3**).

#### Cognitive Challenge Trial

Coefficient of variation of force was predicted by age of the individual for older adults (*P* = 0.01) (step 1 in **Table 3**), but not young adults (*P* > 0.05) (step 1 in **Table 2**). For young adults, step 1 indicated that CV of force during cognitive challenge trial was not predicted by sex or MVC, and in step 2, the Trail-making Test part A or part B was also not predictive (all with *P* > 0.05). In contrast, in the older adults group, each added year in the age of the individual accounted for 0.36%, revealed by the beta value, of the increase in CV of force during the cognitive challenge trial (*P* = 0.01) (step 1 in **Table 3**). The addition of part A of the Trail-making Test improved the regression model for older adults but not young adults (*P* < 0.01 and *P* = 0.65, respectively) (step 2 in **Tables 2**, **3**, respectively). Each unit of increase in *z*-score of the Trail-making Test part A accounted for 0.32% (revealed by the beta value) of the increase in CV of force during cognitive challenge trials for older adults (*P* = 0.01), but not for young adults (*P* = 0.65) (step 2 in **Table 2**). The addition of Trail-making Test part B also improved the regression model for older adults but not young adults (*P* = 0.03 and *P* = 0.20, respectively) (step 2 in **Tables 2**, **3**, respectively). Each *z*-score unit of increase in part B of the Trail-making Test accounted for 0.27% of the increase in CV of force during cognitive challenge trial in older adults (*P* = 0.03), but not young adults (*P* = 0.20).

## Discussion

This study showed that (1) healthy older adults had greater CV of force (i.e., reduced force steadiness) than young adults when a difficult cognitive challenge was imposed during a low-force task with the elbow flexor muscles, (2) young and older women had greater CV of force than men with the imposition of a cognitive challenge, and (3) poor executive functioning, which is a fundamental cognitive ability that allows planning and integration of information during motor tasks, is a significant factor that predicts the age-related decline in force steadiness when a cognitively challenging task is performed simultaneously with a motor task in healthy community-dwelling older adults. To examine the influence of executive functioning on force steadiness, our participants performed the Trail-making Test that largely depends on executive function skills (Sanchez-Cubillo et al., 2009; Camilleri et al., 2015). We found that both part A and B of the Trail-making Test separately improved the prediction of CV of force during a cognitive challenge trial in older adults, but not in the young adults (**Tables 2**, **3**). Parts A and B of the Trail-making Test are highly intercorrelated, so they cannot both be effectively entered into the models. Yet, they are recognized as measuring related but separable constructs (Bowie and Harvey, 2006). Importantly, the influence of executive function on CV of force was independent of age-related changes in cognitive function or education level because we used the *z*-score of each individual Trail-making Test that is adjusted for age and education. We also found that MVC and age of the individual had a greater contribution than the sex of the individual to improve the model to predict CV of force during the cognitive challenge session in young and older adults, but sex was a main factor explaining force steadiness during control contractions of young adults (**Tables 2**, **3**).

### Age and Sex Differences in Force Steadiness During Control and Cognitive Challenging Tasks

Findings of the current study agree with others showing that older adults have greater CV of force (lower force steadiness) during muscle contractions under control conditions when no cognitive challenge is imposed (Enoka et al., 2003; Oomen and van Dieen, 2017, for review). A new finding, however, is that within the older cohort, CV of force for the control contractions had a trend to be even greater in the very older compared with the younger older adults (age factor of step 1 in **Table 3**). Importantly, age-related reductions in force steadiness were further increased when a cognitive challenge was imposed during the force task, particular in women, which is consistent with previous observations for the upper limb muscles (Voelcker-Rehage et al., 2006; Pereira et al., 2015; Shortz and Mehta, 2017) and lower limb muscles (Vanden Noven et al., 2014). Our results showed that prediction of CV of force during cognitive challenge tasks in the older adults group was improved when executive functioning skills (i.e., results from Trail-making Test part A or B) were considered (step 2 in **Table 3**). Together these findings indicate that executive functioning and the age of the individual are primary factors explaining the CV of force during cognitively challenging tasks.

Women were shown in other studies to exhibit a greater CV of force during low-to-moderate force contractions without imposition of a cognitive challenge for the upper limb (Christou et al., 2004; Brown et al., 2010) and lower limb muscles (Vanden Noven et al., 2014). Here we also showed that young and older women had greater CV of force of the elbow flexor muscles during a cognitive challenge as previously reported (Noteboom et al., 2001; Pereira et al., 2015). Furthermore, our current results indicate that during contractions without a cognitive challenge, the sex and strength (MVC) of young adults are primary factors influencing the CV of force (step 1 in **Table 2**), whereas for older adults only the MVC was a main predictor of the force steadiness (step 1 in **Table 3**). There were no sex differences in the Trail-making Test scores for either age group. Thus, sex differences in executive functioning probably do not explain the sex differences in force steadiness.

A larger CV of force is more likely to occur in weaker young and older individuals during control contractions (Brown et al., 2010; Marmon et al., 2011) and our results support these findings (step 1 in **Tables 2**, **3**). However, for a contraction with the cognitive challenge, maximal strength did not improve the model to predict CV of force compared with age of the individual or Trail-making-Test results in young or older adults (step 1 in **Tables 2**, **3**). The findings of age and sex-related differences in CV of force during a cognitive challenge task expand previous observations of impaired motor function during several motor tasks of the upper extremity such as dexterity tests, finger tapping tasks and reaction time tests in young and older adults (Zijdewind et al., 2006; Fraser et al., 2010; Toosizadeh et al., 2016). Physiological mechanisms driving the differences in motor performance during cognitive challenging tasks in men and women are not fully understood, but alterations in cognitive function with aging may partly explain these findings as discussed below.

### Executive Function Influence on Motor Performance

Executive functioning was previously shown to be crucial for *dynamic* contractions such as maintenance of speed in older adults during attention-demanding walking (Ble et al., 2005; Coppin et al., 2006; Springer et al., 2006), a finger tapping task (Fraser et al., 2010), a dexterity task (Corti et al., 2017), and a motor sequencing task with the upper extremity (Niermeyer et al., 2017). The current study shows that the Trail-making Test performance predicted the force steadiness during *static* contractions performed simultaneously with a cognitively challenging task in older adults, but not in young adults. The greater influence of executive functioning on the motor task in the older adults only, may indicate neuroanatomical alterations with aging. More specifically, older adults are known to use additional cortical areas to compensate for the reduced volume of the cortex that is known to occur with aging (Grady, 2012; Reuter-Lorenz and Park, 2014). The mental math performed during the elbow flexion contraction increased the demand of the task by taxing executive function (Menon et al., 2002) and the prefrontal cortex (Baddeley, 2003). Because of the recruitment of additional cortical areas with aging (Grady, 2012; Reuter-Lorenz and Park, 2014), any interference input of the prefrontal cortex on the pre-motor areas (Tanji and Hoshi, 2008) is more likely to occur in older adults. Support for this hypothesis also comes from a greater corticomuscular coherence in the alpha and beta bands of older adults when they perform a mental math task simultaneously with an index finger abduction (Johnson and Shinohara, 2012). However, our data extend the literature to indicate it is those older adults who have greater difficulty with executive function skills who also exhibit the largest impairment in CV of force during cognitive challenge trials. Additionally, all the individuals in the current and previous studies (Ble et al., 2005; Coppin et al., 2006; Springer et al., 2006) were within the range of normal MMSE values (>24), indicating that none of the individuals had severe cognitive impairment. These findings also indicate that older individuals with declines in cognitive processes, although not enough to be considered with a clinical diagnosis, may be at risk of low motor function when performing cognitively challenging motor tasks.

There are other factors that were not tested in this study but that can influence force steadiness in old adults. For example, practice of a force task can improve force steadiness (Laidlaw et al., 1999; Griffin et al., 2009; Onushko et al., 2014) and greater visual feedback was reported to reduce force steadiness particularly in older adults (Christou, 2011; Baweja et al., 2015). Both these factors were not investigated in the current study, although were held constant for both age groups across the different conditions. Other factors such as production of nitrogen and oxygen species and noradrenergic concentration that are known to increase during stressful events (Sessa et al., 2018) such as a cognitively challenging task, may effect young and old adults differently. Reactive oxygen species for example, may impair force production by inhibiting calcium sensitivity (Debold, 2015) although its effects on force steadiness with aging are not known. In contrast, increased noradrenergic concentration may improve force steadiness in young men (Klass et al., 2018), but its effects among older adults is also not understood.

## Conclusion

Healthy community-dwelling older adults, who have lower executive functioning skills, exhibit the greatest reduction of force steadiness (greater CV of force) during an upper extremity motor task when required to perform a cognitively challenging task. Because everyday tasks are frequently performed with the requirement of a dual task involving a cognitive and motor task, healthy community-dwelling older adults, who have poorer executive function skills, are likely at risk of reduced capacity to perform daily activities that involve cognitively challenging motor tasks. Maintenance of executive functioning in older age may impact the performance of steadiness tasks in the presence of greater cognitive demand.

## Author Contributions

HP, SH, and KN designed the study. HP and BS-D collected the data. HP, BS-D, and KN analyzed the data. All authors interpreted the results, contributed to the drafting, and revised the manuscript. SH and KN raised funding for the study.

## Conflict of Interest Statement

The authors declare that the research was conducted in the absence of any commercial or financial relationships that could be construed as a potential conflict of interest.
